# Brain Spectroscopy Analysis in Retired Soccer Players With Chronic Exposure to Mild Traumatic Brain Injuries

**DOI:** 10.1089/neur.2023.0020

**Published:** 2023-08-18

**Authors:** Lucas Lopes Resende, Claudia da Costa Leite, Bruno Fraccini Pastorello, Davi Jorge Fontoura Solla, Pedro Nascimento Martins, Bernardo Fernandes Pelinca da, Mateus Rozalem Aranha, Suely Fazio Ferraciolli, Maria Concepción García Otaduy

**Affiliations:** ^1^ Laboratorio de Ressonancia Magnetica em Neurorradiologia (LIM-44), Instituto e Departamento de Radiologia, Hospital das Clinicas HCFMUSP, Faculdade de Medicina, Universidade de Sao Paulo, Sao Paulo, Brazil.; ^2^ Divisao de Neurocirurgia, Departamento de Neurologia, Faculdade de Medicina FMUSP, Universidade de Sao Paulo, Sao Paulo, Brazil.; ^3^ Faculdade de Medicina, Universidade Federal de Juiz de Fora (UFJF), Juiz de Fora, MG, Brazil.

**Keywords:** brain trauma, magnetic resonance image, soccer, proton magnetic resonance spectroscopy

## Abstract

Soccer players are at risk of suffering cranial injuries in the short and long term. There is growing concern that this may lead to traumatic brain injury in soccer players. Magnetic resonance spectroscopy (MRS) is an analytical method that enables the measurement of changes in brain metabolites that usually occur before significant structural changes. This study aimed to use MRS to compare variations in brain metabolite levels between retired soccer players and a control group. Twenty retired professional soccer players and 22 controls underwent magnetic resonance imaging, including MRS sequences and Mini-Mental State Examination (MMSE). Metabolite analysis was conducted based on absolute concentration and relative ratios. N-acetyl-aspartate, choline, glutamate, glutamine, and myoinositol were the metabolites of interest for the statistical analysis. Retired soccer players had an average age of 57.8 years, whereas the control group had an average age of 63.2 years. Median cognitive evaluation score, assessed using the MMSE, was 28 [26–29] for athletes and 29 [28–30] for controls (*p* = 0.01). Uni- and multi-variate analyses of the absolute concentration of metabolites (mM) between former athletes and controls did not yield any statistically significant results. Comparison of metabolites to creatine ratio concentrations did not yield any statistically significant results. There were no changes in concentrations of brain metabolites that indicated brain metabolic changes in retired soccer players compared with controls.

## Introduction

Soccer is currently the world's most popular sport, with an average of 265 million players worldwide. Soccer players face an increased risk of cranial injuries, primarily attributed to heading maneuvers. Previous research has demonstrated that repeated traumatic brain injury (TBI) can lead to neuropsychological impairment, and there exists a correlation between the frequency of heading strikes and cognitive decline. There is a growing concern regarding the potential development of TBI among soccer players as a consequence of this exposure, which may increase the likelihood of developing neurodegenerative sequelae in this population.^1–4^

Professional American football players experience repeated TBI, which has been associated with chronic traumatic encephalopathy (CTE), a neurodegenerative disease characterized by neuropsychological deficits, compromised executive function, mood alterations, and cognitive dysfunction.^5,6^ The most widely accepted theory links CTE to the progressive deposition of tau-protein neurofilaments in the brain.^7,8^ In addition to abnormalities related to tau protein, there are also reports of rarefaction areas of myelin, neuronal loss, and perivascular hemosiderin deposition.^9^ The majority of investigations exploring the association between athletes and TBI have primarily concentrated on persons engaged in American football as well as other contact sports such as boxing and rugby.

Magnetic resonance spectroscopy (MRS) is an analytical method that enables the detection and measurement of brain metabolites.^10^ Changes in concentrations of these metabolites usually occur before significant structural changes, allowing for earlier and more subtle diagnoses.^11,12^ To date, there is only one published study that used MRS to investigate brain alterations in former soccer players.^13^ They studied a small group of 11 players with no history of concussion and found altered neurochemistry. This highlights the importance of more studies using MRS to assess brain neurometabolism in soccer-related TBI.

In our study, we investigated by MRS a larger and older sample of retired soccer players and compared the results with a control group.

## Methods

### Sampling

The study included 20 retired professional male soccer players and 22 control participants with no history of cognitive dysfunction or TBI. The ethics committee of our institution approved this study.

The inclusion criterion was being a retired professional male soccer player who signed a consent form. Exclusion criteria were previous TBI unrelated to soccer, an absolute contraindication to magnetic resonance imaging (MRI) examinations, image artifacts that made it difficult to process images with segmentation and quantification software, inadequate MRS analysis, or detection of other brain pathologies through MRI.

All soccer players included in this study had a history of extensively heading the ball during their professional practice. The number of heading instances they experienced was significant and incalculable. Table 1 provides a comprehensive overview of demographic and clinical data. For comparison, the age-matched control group consisted of males with no reported history of TBI or concussions.

**Table 1.** Demographic and Clinical Data from Soccer Players and Controls

***Characteristic***	***Athletes,*** *N* *** = 20* ^a^**	***Controls,*** *N* *** = 22* ^a^**	*p* ***value* ^b^**
Age, years	57.8 ± 10.8	63.2 ± 13.7	0.159
Level of education	12.6 ± 2.9	14.7 ± 2.7	**0.017**
MMSE	28 [26–29]	29 [28–30]	**0.010**
Duration of career in years	19.6 ± 6.2	—	
TBI	10 (50)	—	
Concussion	7 (35)	—	
Type of impact			
Head to head	8 (80)	—	
Head to head and head to ball	1 (10)	—	
Head to ground	1 (10)	—	
Player position			
Defense	11 (55)	—	
Midfield	6 (30)	—	
Forward	3 (15)	—	

^a^ Mean ± SD; median [IQR]; *n* (%).

^b^ Results obtained by *t*-test and Wilcoxon's rank-sum test.

TBI, traumatic brain injury; MMSE, Mini-Mental State Examination; SD, standard deviation; IQR, interquartile range .

Participants underwent screening for cognitive impairment using the MMSE.

### Image acquisition

All subjects underwent MRI using a specific coil for an eight-channel head on a 3.0 Tesla magnet (PET-MRI/GE Discovery MR 750). Each brain examination included three-dimensional (3D) T1-weighted fluid-attenuated inversion recovery (FLAIR), magnetic susceptibility sequences, and MRS. FLAIR, T1, and magnetic susceptibility images were analyzed qualitatively to exclude lesions unrelated to mild TBI, leaving only lesions related to CTE and habitual aging. None of the subjects were excluded from the study because of lesions.

**FIG. 1.**
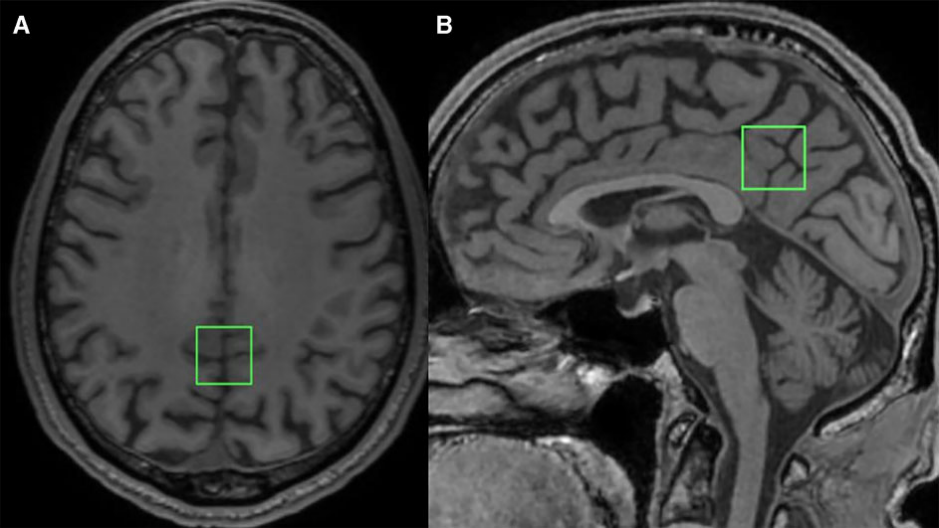
Graphical representation of voxel position in the posterior cingulate gyrus in a T1-weighted 2D image. Axial (**A**) and sagittal (**B**) views. 2D, two-dimensional .

The voxel used to evaluate MRS was placed on the posterior cingulate gyrus, planned on a T1-weighted 3D image with dimensions of 20 × 20 × 20 mm, resulting in an 8-cm³ cube (Fig. 1). Placement of the MRS region of interest (ROI) was verified by an experienced, board-certified neuroradiologist. The single-voxel spectroscopy sequence was performed using point-resolved spectroscopy with a 1500-ms repetition time, 35-ms echo time (TE), 5000-Hz receiver bandwidth, 4096 spectral points, and 128 excitation numbers, including an additional acquisition of two water non-suppressed spectra for reference purposes. The MRS sequence was preceded by automatic pre-acquisition, including receiver-transmitter adjustment, scanner default water suppression, and field-homogeneity adjustment of the chosen voxel. After pre-scan, the calculated full width at half maximum (FWHM) of the spectrum was observed. For all cases, this value was <12 Hz and there was no need for voxel replacement.

### Magnetic rsonance spectroscopy quantification

**FIG. 2.**
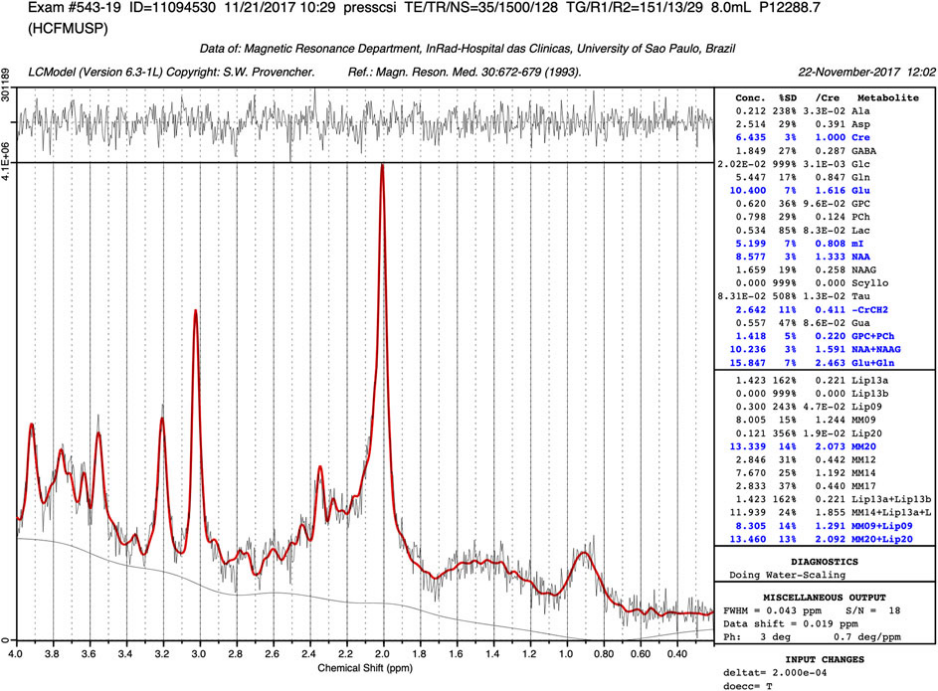
Spectral curve model obtained by spectroscopy data processing in LCModel software.

Spectroscopic data were processed and analyzed using Linear Combination Model (LC Model/Version 6.1) software standard analysis. The program provided absolute values relative to the water content and ratios of metabolites to creatine (Fig. 2). LCModel processing also provides measures of signal-to-noise ratio (S/N) and spectral linewidth (FWHM) for objective quality assessment.

For statistical analysis, we calculated metabolite ratios relative to the creatine (Cr) concentration, including N-acetyl-aspartate (NAA), choline (Cho), glutamate (Glu), glutamine (Gln), and myoinositol (mI). Because normal metabolic concentrations vary considerably between gray matter (GM) and white matter (WM),^14^ we needed to consider the fraction of WM in the voxel during the analysis. We used Gannet software^15^ to co-register the MRS voxel with the 3D T1-weighted brain image and calculate the GM, WM, and cerebrospinal fluid (CSF) percentage in the voxel based on SPM^16^ segmentation of the T1-weighted image. The WM brain tissue fraction (fWM) was calculated for each voxel (fWM = %WM/[%GM +%WM]), and fWM was used as a covariate when comparing relative metabolite concentrations between groups.

We used voxel water content as a reference to estimate absolute metabolite concentrations in mM, as described by Gasparovic and colleagues.^14^ Tissue segmentation within the voxel was performed in order to take into account the different water content in WM, GM, and CSF (36.11, 43.33, and 53.89 M, respectively), as well as the different relaxation properties of water in CSF (T1 = 4.16 sec, T2 = 0.5 sec), GM (T1 = 1.82 sec, T2 = 0.10 sec), and WM (T1 = 1.08 sec, T2 = 0.07 sec). For T1, metabolites of NAA, Glu, and Glx T1 were assumed as 1.47, 1.27, and 1.20 sec in GM and as 1.35, 1.17, and 0.96 sec in WM, respectively.^17^ For NAA, T2 values of 0.247 and 0.295 sec in the GM and WM were used, respectively. For Glu and Glx, the T2 value of 0.2 sec was assumed.^18^

### Statistical analysis

Participants' age and level of education were reported using means and standard deviations and compared between groups using a *t*-test. MMSE scores were described using the median and interquartile range because of their asymmetric distribution caused by the notable ceiling effect. Consequently, a Wilcoxon test was used to compare the scores. Metabolites and MRS parameters analyzed by spectroscopy were found to have a Gaussian distribution. They were described using means and standard deviations and compared using a *t*-test. Multi-variate analysis and linear models were used to compare absolute and relative metabolite concentrations between groups, taking into account age and level of education for absolute concentration analysis. WM fraction was included in the multi-variate analysis along with age and level of education only to compare metabolite to creatine ratios. Education level was considered a confounding variable, and given its importance, two missing values were obtained by imputing the average values. Statistical analysis was conducted using R software (2022; R Foundation for Statistical Computing, Vienna, Austria).

## Results

### Sociodemographic and clinical clinical data

A total of 42 participants were included in the analysis: 20 former soccer players and 22 controls. Table 1 summarizes their sociodemographic and clinical data. Participants had a mean age of 60.6 ± 12.53 years, with former soccer players having a mean age of 57.8 ± 10.77 years and controls having a mean age of 63.2 ± 13.67 years (*p* = 0.159).

Controls had a higher level of education than former soccer players, with the former group averaging 14.75 ± 2.70 years and the latter averaging 13.60 ± 2.89 years (*p* = 0.017). Median cognitive evaluation score, assessed using the MMSE, was 28 [26–29] for athletes and 29 [28–30] for controls (*p* = 0.01).

Among athletes, mean duration of their careers was 19.6 ± 6.2 years. Athletes playing in defense accounted for 55% of our cohort, midfielders 30%, and forwards 15%. Approximately 50% of athletes reported experiencing a TBI during their career. Regarding the types of impacts, head-to-head collisions were reported by 80% of athletes, head-to-head and head-to-ball impacts by 10%, and head-to-ground impacts by 10%. Following the classification by McCrory and colleagues, sports-related concussions typically result in the rapid onset of transient neurological impairment and a variety of clinical signs and symptoms, which may or may not involve loss of consciousness.^19^ Therefore, in our cohort, patients who reported TBIs associated with any neurological symptoms were classified as having experienced a concussive event. Among athletes, 35% had a concussion.

### Magnetic resonance spectroscopy

Table 2 provides detailed information on spectroscopy quality and voxel segmentation data. No significant disparity in S/N and FWHM values was observed between athletes and controls. Metabolite mean Cramer-Rao lower bound (CRLB) values were also not different for both groups, as shown in Table 3. Athletes had a higher average WM percentage (18% ± 6%) than controls (14% ± 4%; *p* = 0.032). Percentages of GM and CSF did not differ significantly between the two groups, with controls averaging 61% ± 6% and 25% ± 8%, respectively, whereas athletes averaged 58% ± 8% and 24% ± 8%, respectively.

**Table 2.** Magnetic Resonance Spectroscopy Data Details

***Data***	***Athletes*^a^ *(***n*** = 20)***	***Controls*^a^ *(***n*** = 22)***	*p* ***value* ^b^**
FWHM, ppm	0.04 ± 0.01	0.04 ± 0.01	0.941
S/N	20.10 ± 3.54	21.05 ± 3.04	0.365
Voxel	*n* = 20	*n* = 17	
%WM	0.18 ± 0.06	0.14 ± 0.04	0.032
%GM	0.58 ± 0.08	0.61 ± 0.06	0.176
%CSF	0.24 ± 0.08	0.25 ± 0.08	0.754

^a^ Mean ± SD.

^b^ Results obtained by *t*-test.

FWHM, full width at half maximum; S/N, signal-to-noise ratio; %WM, white-matter percentage; %GM, gray-matter percentage; %CSF, cerebrospinal fluid percentage; ppm, parts per million; SD, standard deviation.

**Table 3.** Uni- and Multi-Variate Analysis of Brain Metabolites Adjusted for Age and Education

***Metabolites***	***Athletes (*** *N* *** = 20)***	***CRLB***	***Controls (*** *N* *** = 17)***	***CRLB***	*p* ***value***	***Adj. Diff***	*p* ***value (adj)***
Cre	7.25 ± 0.91	2.70 ± 0.47	7.46 ± 0.51	2.48 ± 0.50	0.383	0.19 (−0.29 to 0.67)	0.444
Cho	1.22 ± 0.14	5.00 ± 0.78	1.30 ± 0.13	5.33 ± 1.50	0.055	0.07 (−0.02 to 0.16)	0.131
Glu	8.45 ± 1.31	6.60 ± 0.75	8.91 ± 0.68	6.20 ± 1.00	0.179	0.29 (−0.43 to 1.00)	0.439
Glx	11.77 ± 2.01	6.70 ± 0.98	11.95 ± 0.99	6.62 ± 1.05	0.721	0.00 (−1.1 to 1.1)	0.999
mI	4.79 ± 0.62	5.00 ± 0.79	4.91 ± 0.52	5.10 ± 1.53	0.503	0.12 (−0.25 to 0.49)	0.526
NAA	8.47 ± 1.05	3.00 ± 0.56	8.43 ± 0.56	2.90 ± 0.22	0.888	–0.17 (−0.74 to 0.39)	0.550

Results obtained by the *t*-test.

A = athlete; C = control.

CRLB, Cramer-Rao lower bound; Cre, creatine; Cho, choline; Glu, glutamate; Glx, glutamine + glutamate; mI, myoinositol; NAA, N-acetyl aspartate; Adj.Diff., adjusted difference.

### Analysis of absolute metabolite concentrations

**FIG. 3.**
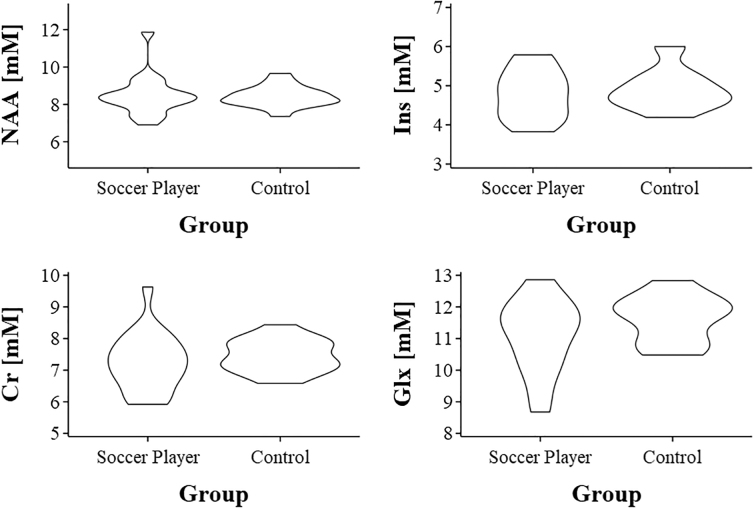
Violin plot of metabolite absolute concentration (mM) distribution according to groups. Cr, creatine; Glx, glutamine + glutamate; Ins, inositol; NAA, N-acetyl aspartate.

Table 3 displays the results of the uni- and multi-variate analyses of the absolute concentrations of metabolites (in mM) between former athletes and controls. Creatine concentration was slightly higher in controls (7.46 ± 0.51 mM) than in former soccer players (7.25 ± 0.91 mM). The absolute level of choline was also slightly higher in controls (1.3 ± 0.13 mM) than in former soccer players (1.22 ± 0.14 mM). The absolute level of NAA was similar in both groups, with controls having 8.43 ± 0.56 mM and former soccer players having 8.47 ± 1.05 mM (Fig. 3). These differences were not statistically significant, and the distributions of the absolute concentrations are shown in Figure 3. After adjusting for possible confounding factors, there was a lower NAA concentration in athletes than in controls (0.17; 95% CI, 0.74–0.39), but this difference was not statistically significant (*p* = 0.550). Concentrations of other metabolites were higher in controls than in athletes; however, none showed statistically significant differences.

### Relative metabolites concentration analysis

**FIG. 4.**
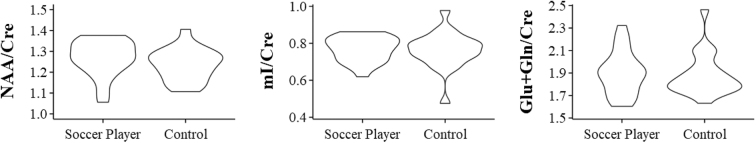
Violin plot of metabolite relative concentration distribution according to groups. Cre, creatine; mI, myoinositol; Gln, glutamine; Glu, glutamate; MM, macromolecules; NAA, N-acetyl aspartate.

Table 4 shows the results of uni- and multi-variate analyses of metabolite concentrations relative to creatine, adjusted for age, level of education, and WM percentage. NAA to creatine ratio was similar in both groups, with controls having 1.24 ± 0.08 and former soccer players having 1.27 ± 0.09 (*p* = 0.125). Similar to the previous analysis of the absolute metabolite concentrations, no statistically significant differences were observed (Fig. 4). In multi-variate analysis, athletes had higher concentrations of NAA, Gln, Glx, and ml, but the difference was not statistically significant (*p* > 0.05).

**Table 4.** Uni- and Multi-Variate Analysis of Brain Metabolites to Creatine Ratios Adjusted for Age, Education, and White Matter Fraction

***Metabolites***	***Athletes (*** *N* *** = 20)***	***Control (*** *N* *** = 22)***	*p* ***value***	***Adj. Diff (*** *N* ***: A = 20/C = 17)***	*p* ***value (adj)***
Gln/Cre	0.58 ± 0.15	0.53 ± 0.11	0.239	0.06 (−0.05 to 0.17)	0.288
Glu/Cre	1.32 ± 0.13	1.35 ± 0.11	0.401	–0.02 (−0.11 to 0.07)	0.723
(Glu + Gln)/Cre	1.90 ± 0.19	1.88 ± 0.19	0.780	0.04 (−0.11 to 0.19)	0.594
mI/Cre	0.77 ± 0.07	0.76 ± 0.10	0.828	0.01 (−0.05 to 0.06)	0.834
NAA/Cre	1.27 ± 0.09	1.24 ± 0.08	0.211	0.04 (−0.01 to 0.10)	0.125
Cho/Cre	0.17 ± 0.02	0.18 ± 0.02	0.315	–0.01 (−0.02, 0.01)	0.454

Results obtained by the t-test.

A = athlete; C = control.

Cre, creatine; Cho, choline; Glu, glutamate; Glx, glutamine + glutamate; mI, myoinositol; NAA, N-acetyl aspartate; Adj.Diff., adjusted difference.

## Discussion

Soccer players are at risk for various types of TBI both in the short and long term.^20^ Studies have shown that recurrent TBI may result in neuropsychological damage, with the number of heading strikes being linked to cognitive decline.^21,22^ Further, soccer athletes have been found to experience a significant number of concussions throughout their careers.^5,23^

This particular study analyzed a total of 42 subjects, with the average age of retired soccer players and controls being 57.8 and 63.2 years, respectively. Although the controls had an average of 2.15 years more schooling than the retired soccer players, statistical analysis using linear regression models accounted for this potential confounding variable, making false-negative outcomes less likely. The cognitive analysis using the MMSE revealed a median score of 28 [26–29] for athletes and 29 [28–30] for controls (*p* = 0.01). Both groups exhibited a prominent ceiling effect. Therefore, despite the statistically significant between-group difference, it lacked clinical relevance. However, it is well documented that soccer players have an increased susceptibility to developing neurological and neurocognitive impairments in later stages of life.^24^ In the current study, despite this knowledge, the neurocognitive functioning of soccer players was found to be within the normal range.

The athletes exhibited a higher proportion of WM in the voxels analyzed, whereas GM and CSF showed no statistically significant differences. To account for potential confounding factors, the linear regression model was supplemented with the WM fraction in the multi-variate analysis for metabolite-to-creatine ratios. McLean and colleagues recommended estimating the distribution of WM, GM, and CSF voxels to correct for the likely diluting effects of CSF and metabolic differences between WM and GM. Additionally, normal metabolite concentrations vary between the WM and GM, which can also be confounding factors.^25^

Examination of brain metabolites did not reveal any significant differences in their concentrations. Only one study has investigated changes in brain metabolism in soccer players using MRS. For instance, Koerte and colleagues compared spectroscopic data from 11 former soccer players and 15 age-matched controls (ages 40–70 years). The results of this study indicated a notable increase in Cho (a biomarker for the cell membrane) and ml (a biomarker for glial activation). The present study, which had a larger sample size than the previously published studies, used a short TE (35 ms) to identify and measure more metabolites. However, the differences in the Cho and mI concentrations were not statistically significant. In contrast to Koerte and colleagues, this study used multi-variate analysis that accounted for potential confounding factors in the model. The absence of information on possible confounding factors in Koerte and colleagues' study may explain the discrepancies with existing literature. Another difference between the current study and that of Koerte and colleagues is the type of sample. Half of the retired athletes in our cohort reported a history of sports-related TBI, whereas in Koerte and colleagues' study, athletes did not have a history of concussion.^13^

Most studies of the relationship between athletes and TBI have focused on American football players. Alosco and colleagues conducted a study in which they analyzed brain metabolites in former American football players and compared them to asymptomatic controls who had not experienced previous TBI. They found a significant decrease in concentrations of NAA and Cr in these players, suggesting a decrease in neuronal energy metabolism in the brain.^26^ Another study, which had a smaller sample size of 5 players with an average age of 43.6 years, analyzed neurochemical changes in the brain using spectroscopy. This study showed increased levels of glutamate/glutamine and choline, whereas NAA and mI markers did not exhibit statistically significant differences.^27^

A study that compared 16 retired rugby players (ages 30–45) who had experienced concussions during their sports practice with healthy controls found similar results. Although rugby involves more intense trauma than soccer, no significant decreases in NAA, mI, Cho, or Glu levels were observed in this study.^28^

Studies reporting repeated mild TBI have shown reductions in NAA concentrations, which is a biomarker for neuronal viability, and changes in Cho concentrations, which is a biomarker of tissue damage and cellular proliferation. These findings suggested that chronic exposure to TBI results in definitive neuronal loss and axonal damage.^29–31^ Our analysis of former soccer players did not uncover statistically significant alterations in NAA and Cho levels. This finding suggests a potential absence of neurodegeneration, given that levels of NAA, a marker of neuronal viability, were found to be similar in both groups. Preservation of NAA levels in the studied players may be attributed to the possibility that they have not yet surpassed the threshold for neuronal degeneration. Concentrations of the remaining analyzed metabolites did not exhibit a statistically significant difference between former soccer players and controls. This indicated that there may not have been any metabolic variation in the brain between the two groups in the given sample.

The posterior cingulate gyrus was selected as the ROI for spectroscopy in this study for several reasons. First, it is frequently chosen in MRI brain spectroscopy investigations related to TBI.^32^ Previous TBI studies, including those involving soccer athletes, have utilized this region, making it a valuable point of comparison for the current subjects.^25^ Additionally, this region is highly homogeneous, ensuring excellent technical quality and reproducibility during MRI examinations. It is also less susceptible to artifacts, enabling reliable data collection. Although adding a predominantly WM ROI would have been beneficial, it was not feasible to collect more data in our cohort because of time constraints.

However, this study has some limitations. First, the sample size was small, although it is larger than that of previous studies that examined the brains of former soccer players using proton MRS. Additionally, some data could have been gathered for a more detailed analysis, such as information on alcohol consumption, estimated frequency of heading during their careers, lifestyle factors, and psychosocial factors that influence WM content of the brain. This study focused on the posterior cingulate GM; therefore, we must acknowledge our inability to extrapolate our results to the WM, and the potential presence of WM injury cannot be ruled out. Further, inferences about the whole brain should not be made based on neurometabolite alteration in one region. The results of this study need to be confirmed in future studies using larger sample sizes and more comprehensive data.

## Conclusion

Concentration levels of brain metabolites did not reveal any significant changes that could indicate metabolic alterations in retired soccer players compared with controls.

## Abbreviations Used

Cho: choline
CRLB: Cramer-Rao lower bound
CSF: cerebrospinal fluid
CTE: chronic traumatic encephalopathy
FLAIR: fluid-attenuated inversion recovery
FWHM: full width at half maximum
fWM: WM brain tissue fraction
Gln: glutamine
Glu: glutamate
GM: gray matter
mI: myoinositol
MMSE: Mini-Mental State Examination
MRI: magnetic resonance imaging
MRS: magnetic resonance spectroscopy
NAA: N-acetyl aspartate
ROI: region of interest
S/N: signal-to-noise ratio
TBI: traumatic brain injury
TE: echo time
WM: white matter
